# The clinical implication of gamma-glutamyl transpeptidase in COVID-19^☆^

**DOI:** 10.1016/j.livres.2021.09.001

**Published:** 2021-09-25

**Authors:** Jianrong Liu, Chao Yu, Qing Yang, Xiaofeng Yuan, Fan Yang, Panlong Li, Guihua Chen, Weicheng Liang, Yang Yang

**Affiliations:** a Department of Hepatic Surgery and Liver Transplantation Center, The Third Affiliated Hospital of Sun Yat-sen University, Guangzhou, China; b Surgical and Transplant Intensive Care Unit, The Third Affiliated Hospital of Sun Yat-sen University, Guangzhou, China; c Cell-gene Therapy Translational Medicine Research Center, The Third Affiliated Hospital of Sun Yat-sen University, Guangzhou, China; d Medical Examination Center, The Third Affiliated Hospital of Sun Yat-sen University, Guangzhou, China; e Organ Transplantation Research Center of Guangdong Province, The Third Affiliated Hospital of Sun Yat-sen University, Guangzhou, China; f Biotherapy Center, The Third Affiliated Hospital of Sun Yat-sen University, Guangzhou, China; g Department of Infectious Diseases, The People's Hospital of Kashgar, Kashgar, Xinjiang, China; h Department of Infectious Diseases, Xinjiang Medical University, Urumqi, China; i Organ Transplantation Institute of Sun Yat-sen University, The Third Affiliated Hospital of Sun Yat-sen University, Guangzhou, China; j Guangdong Province Engineering Laboratory for Transplantation Medicine, The Third Affiliated Hospital of Sun Yat-sen University, Guangzhou, China

**Keywords:** Coronavirus disease 2019 (COVID-19), Severe acute respiratory syndrome coronavirus 2 (SARS-CoV-2), Gamma-glutamyl transpeptidase (GGT), Angiotensin-converting enzyme 2 (ACE2), Liver injury, Bile duct injury

## Abstract

**Background and aim:**

Coronavirus disease 2019 (COVID-19) is a life-threatening disease that predominantly causes respiratory failure. The impact of COVID-19 on other organs remains elusive. Herein, we aimed to investigate the effects of COVID-19 on the hepatobiliary system.

**Methods:**

In the current study, we obtained the clinical records and laboratory results from 66 laboratory-confirmed patients with COVID-19 at the Wuhan Tongji Hospital between 10 February 2020 and 28 February 2020. The detailed clinical features and laboratory findings were collected for analysis. Bioinformatics analysis was conducted to evaluate the correlation between gamma-glutamyl transferase (GGT) and severe acute respiratory syndrome coronavirus 2 (SARS-CoV-2) entry receptor angiotensin-converting enzyme 2 (ACE2).

**Results:**

In this cohort, 30 (51.7%) patients had abnormal liver function on admission, which was associated with disease severity and enriched in the male and diabetic patients. The elevated levels of direct bilirubin (*P* = 0.029) and GGT (*P* = 0.004) were common in patients with severe pneumonia when compared with those with mild pneumonia. In addition, elevated levels of GGT (*P* = 0.003) and aspartate aminotransferase (AST) (*P* = 0.007) were positively associated with longer hospital stay. The expression of ACE2 was closely associated with GGT in various human tissues because they shared the common transcriptional regulator hepatic nuclear factor-1β (HNF1B).

**Conclusions:**

Increased GGT levels were common in severe cases and elevated GGT levels were positively associated with prolonged hospital stay and disease severity. Due to the consistent expression with ACE2, GGT is a potent biomarker indicating the susceptibility of SARS-CoV-2 infection.

## 1 Introduction

As of 4 August 2021, coronavirus disease 2019 (COVID-19) caused by the severe acute respiratory syndrome coronavirus 2 (SARS-CoV-2) had been responsible for approximately 197.8 million infections worldwide, according to the World Health Organization (WHO) COVID-19 weekly situation report. The number of COVID-19 cases has reached a pandemic level. Its deleterious impact posing on society calls for a rapid and intensive study of the virus and disease. The most common initial symptoms are fever, cough, myalgia, or fatigue. Sputum production, headache, hemoptysis, and diarrhea could also be found in some patients.^1^, ^2^, ^3^ According to a recent retrospective study, it was found that most of the patients with COVID-19 died of multiple organ failure, including respiratory failure, acute cardiac injury, and acute kidney injury.^4^ Among the reported comorbidities in the victims, sepsis was the most common one, followed by respiratory failure, acute respiratory distress syndrome (ARDS), heart failure, and septic shock. However, the impact of SARS-CoV-2 on the hepatobiliary system remains largely unknown.^5^

Based on the results of high-throughput sequencing, it was found that SARS-CoV-2 shared a 79% sequence similarity with SARS-CoV and a 50% sequence similarity with the Middle East respiratory syndrome coronavirus (MERS-CoV).^6^ It was reported that both SARS and MERS patients were associated with liver function impairment.^7^, ^8^, ^9^ Recently, serial large-scale retrospective studies have reported the clinical features of patients infected with the SARS-CoV-2 virus. According to these studies, it was estimated that 2–11% of patients with COVID-19 had pre-existing liver diseases and 16.1–53.1% of the infected patients had elevated levels of alanine aminotransferase (ALT) and aspartate aminotransferase (AST).^6^ Moreover, compared to patients with nonsevere COVID-19, patients with severe COVID-19 seemed to have a higher incidence of liver dysfunction. In a cohort from Wuhan, Huang *et al.*^2^ reported that elevated AST levels were observed in 62% of patients with COVID-19 in the intensive care unit (ICU). Furthermore, in a large-scale study involving 1099 patients from 552 hospitals in China, patients with severe COVID-19 had a higher percentage of abnormal liver aminotransferase levels than patients with mild COVID-19.^1^

It is known that the distribution of viral receptors might determine the route of SARS-CoV-2 infection, which may account for liver injury in some infected patients. Current advances have highlighted an important role of angiotensin-converting enzyme 2 (ACE2) receptor in mediating the cellular entry of SARS-CoV as well as that of SARS-CoV-2.^10^^,^^11^ The systematic investigations by using single-cell RNA sequencing (scRNA-seq) have illuminated the distribution of ACE2 in various human organs and distinct cell populations within these organs.^12^, ^13^, ^14^, ^15^ Using the supervised graph-based clustering method, the scRNA-seq found that ACE2 was highly expressed in cholangiocytes rather than hepatocytes.^14^ Previous studies showed that SARS-CoV-2 RNA was detectable in the blood samples of COVID-19 patients. Thus, the impact of the SARS-CoV-2 virus on the hepatobiliary system needs to be further investigated.

In the current study, we found that elevated levels of AST, ALT, and gamma-glutamyl transferase (GGT) were frequently present in COVID-19 patients. The levels of direct bilirubin and GGT were significantly higher in patients with severe pneumonia than in those with mild pneumonia. We also found that elevated levels of GGT and AST were positively correlated with a longer hospital stay. Furthermore, we also found that the expression of GGT was closely associated with ACE2 due to the same regulatory network, suggesting that patients with elevated GGT levels may be more susceptible to SARS-CoV-2. Taken together, these results highlighted a previously unknown role of GGT in COVID-19, which may shed light on the prevention of acute liver failure in COVID-19 patients.

## 2 Methods

### 2.1 Ethical approval

This study was approved by the Ethics Committee of Wuhan Tongji Hospital and The Third Affiliated Hospital of Sun Yat-sen University. This study was performed in accordance with the ethical standards laid down in the 1964 Declaration of Helsinki and its later amendments.

### 2.2 Study design and participants

In this retrospective study, we enrolled 66 patients with confirmed COVID-19 who were admitted to the Wuhan Tongji Hospital between 10 February 2020 and 28 February 2020. The presence of SARS-CoV-2 was confirmed by reverse transcriptase-polymerase chain reaction (RT-PCR) in the laboratory. In this cohort, as the presence of some liver diseases may interfere with the results of the liver test, our study excluded eight patients with at least one of the following diseases: hepatitis B virus (HBV), hepatitis C virus (HCV) infection, autoimmune hepatitis, primary biliary cholangitis, and other viral pathogens coinfection (human immunodeficiency virus or *Treponema pallidum*).

### 2.3 Data collection and evaluation

The clinical, laboratory, complications, management, and outcome data were obtained from patients' medical records or direct communication with the patients. The medical records were obtained, including smoking status, onset symptoms, comorbidities, and medication histories. Each patient's axillary temperature was also recorded. Fever was defined as a body temperature higher than 37.5 °C. The laboratory tests were conducted within 24 h of admission, including a complete blood count, coagulation, glucose, liver function tests, cardiac injury biomarkers, procalcitonin, as well as measurements for pro-inflammatory cytokines (interleukin (IL)-6 and tumor necrosis factor-alpha (TNF-α)).

Diabetes was defined as fasting glucose ≥ 7 mmol/L or an existing diagnosis of diabetes mellitus. The clinical outcome of interest was the duration of hospital stay. The duration of stay was defined as the time between admission and discharge. The decision to discharge was based on the abatement of fever and respiratory symptoms, improvement of chest computed tomography evaluation, as well as clear evidence of viral clearance in respiratory samples. Death and a diagnosis of severe pneumonia were also recorded.

Abnormal liver function was defined as an elevation of the serum levels of the following liver enzymes: ALT >33 U/L, AST >32 U/L, GGT >42 U/L, alkaline phosphatase (ALP) >105 U/L, and total bilirubin (TBIL) >21 μmol/L.

### 2.4 Severity of COVID-19

We defined the degree of severity of COVID-19 (mild *vs*. severe) on admission according to the WHO interim guidance for COVID-19 (version 1.2).^16^ Severe pneumonia was diagnosed using the criteria that display at least one of the following conditions: (i) respiratory distress, respiratory rate ≥30 times/min); (ii) oxygen saturation (resting state) ≤93%; (iii) partial pressure of oxygen/fraction of inspired oxygen (PaO_2_)/FiO_2_) ≤300 mm Hg (serious); (iv) the occurrence of respiratory or other organ failures which require ICU monitoring and treatment, or shock. The clinical outcomes, including discharges and length of stay, were followed up until 21 March 2020.

### 2.5 Bioinformatics analysis

The chromatin immunoprecipitation sequencing (ChIP-seq) data of hepatic nuclear factor-1beta (HNF1B) was extracted from the Cistrome database (http://cistrome.org/db/#/). The expression profiles of HNF1B, ACE2, and GGT1 were obtained from GEPIA (Gene Expression Profiling Interactive Analysis, http://gepia.cancer-pku.cn/) and NCBI (National Center of Biotechnology Information, https://www.ncbi.nlm.nih.gov/).

### 2.6 Statistical analysis

Continuous data are presented as means ± standard deviation (SD) (for normally distributed data) and median (interquartile range (IQR)) (for data with skewed distributions). Categorical data are presented as counts and percentages. Categorical variables were tested for differences using the χ^2^ test or Fisher's exact test depending on the category cell size, whereas a two-sample *t*-test or the Mann–Whitney *U* test was used for continuous variables where appropriate. The Kaplan–Meier method was utilized to evaluate the differences in the duration of stay. No adjustments were made for multiple testing. All *P*-values in this study are two-sided. The threshold for statistical significance was set at *P* < 0.05. Statistical analyses were performed using STATA 14.0 (Stata Corporation, USA).

## 3 Results

### 3.1 Clinical features of patients with COVID-19 with and without abnormal liver function on admission

A total of 58 patients were enrolled in this study. Among these patients, 30 (51.7%) were males (Table 1). The average age was 65.1 years. Diabetes (31.0%), coronary artery disease (20.7%), and kidney disease (15.5%) were the most common coexisting diseases. The common symptoms at illness onset were fever (70.7%), cough (62.1%), anorexia (34.5%), fatigue (32.8%), chest tightness (27.6%), and dyspnea (25.9%) (Table 2).

**Table 1:** Clinical characteristics of the patients with COVID-19.

Variable	All (*N* = 58)	With normal liver function (*n* = 28)	With abnormal liver function (*n* = 30)	*P*-value
Age (years)	65.1 ± 14.2	65.7 ± 13.5	64.5 ± 15.0	0.374
Male, *n* (%)	30 (51.7)	9 (32.1)	21 (70.0)	0.004
Current smoker, *n* (%)	8 (13.8)	1 (3.6)	7 (23.3)	0.053
The highest temperature (°C)	37.9 (37.0–38.7)	37.4 (36.5–38.0)	38.3 (37.8–39.0)	0.007
Severe pneumonia, *n* (%)	12 (20.7)	2 (7.1)	10 (33.3)	0.014
Cardiac injury, *n* (%)	8 (13.8)	2 (7.1)	6 (20.0)	0.156
Liver functions				
AST (U/L)	20 (18–27)	19 (18–21)	27 (19–54)	< 0.001
ALT (U/L)	20 (13–36)	13 (11–16)	36 (22–62)	< 0.001
GGT (U/L)	26 (16–51)	17 (12–23)	51 (31–85)	< 0.001
ALP (U/L)	64 (54–75)	56 (52–67)	72 (61–85)	0.001
TBIL (μmol/L)	9.2 (6.6–13.3)	7.5 (5.9–10.2)	10.5 (8.0–19.7)	0.010
DBIL (μmol/L)	3.9 (2.7–5.8)	3.4 (2.4–4.2)	4.5 (3.6–7.7)	0.010
Albumin (g/L)	37.6 (33.8–40.0)	38.7 (35.5–41.1)	35.9 (31.6–38.8)	0.059
Coagulation function				
PT (s)	13.4 (13.0–14.2)	13.3 (12.9–14.2)	13.5 (13.1–14.5)	0.378
PT-INR	1.02 (0.99–1.11)	1.02 (0.98–1.11)	1.04 (1.00–1.14)	0.378
APTT (s)	38.5 (35.9–41.4)	38.2 (36.5–40.6)	39.0 (35.1–42.4)	0.895
D-dimer (mg/L)	0.93 (0.35–2.00)	0.38 (0.25–1.29)	1.45 (0.71–2.89)	< 0.001
Inflammatory markers^a^				
hs-CRP (mg/L)	10.6 (1.3–25.7)	4.2 (0.6–15.3)	13.5 (5.2–57.4)	0.003
IL-6 (ng/L)	4.54 (1.86–11.99)	2.94 (1.69–9.03)	7.61 (2.59–14.24)	0.070
TNF-α (ng/L)	7.7 (5.4–11.9)	6.1 (5.2–7.7)	10.4 (8.6–13.3)	0.003

Data are shown as means ± standard deviation (SD), *n* (%) or median (IQR).

Abbreviations: ALP, alkaline phosphatase; ALT, alanine aminotransferase; APTT, activated partial thromboplastin time; AST, aspartate aminotransferase; COVID-19, coronavirus disease 2019; DBIL, direct bilirubin; GGT, gamma-glutamyl transferase; hs-CRP, high sensitive C-reactive protein; IL-6, interleukin-6; INR, international normalized ratio; IQR, interquartile range; PT, prothrombin time; TBIL, total bilirubin; TNF-α, tumor necrosis factor-alpha; ULN, upper limit of normal.

a Data available for 46 patients. Twenty-four with normal liver function; and 22 with abnormal liver function, respectively.

**Table 2:** Comorbidities and main symptoms of the patients with COVID-19.

Variable	All (*N* = 58)	With normal liver function (*n* = 28)	With abnormal liver function (*n* = 30)	*P*-value
Comorbidities				
Diabetes	18 (31.0)	5 (17.9)	13 (43.3)	0.036
Coronary artery disease	12 (20.7)	4 (14.3)	8 (26.7)	0.336
Cerebrovascular disease	3 (5.2)	1 (3.6)	2 (6.7)	1.000
Kidney disease	9 (15.5)	4 (14.3)	5 (16.7)	1.000
Malignancy	4 (6.9)	1 (3.6)	3 (10.0)	0.612
Chronic obstructive pulmonary disease	1 (1.7)	0	1 (3.3)	1.000
Main symptoms				
Fever	41 (70.7)	17 (60.7)	24 (80.0)	0.107
Cough	36 (62.1)	19 (67.9)	17 (56.7)	0.380
Fatigue	19 (32.8)	10 (35.7)	9 (30.0)	0.643
Headache	5 (8.6)	2 (7.1)	3 (10.0)	0.698
Pharyngalgia	1 (1.7)	1 (3.6)	0	0.296
Myalgia	3 (5.2)	2 (7.1)	1 (3.3)	0.513
Dizziness	1 (1.7)	0	1 (3.3)	0.330
Chest tightness	16 (27.6)	8 (28.6)	8 (26.7)	0.871
Dyspnea	15 (25.9)	6 (21.4)	9 (30.0)	0.456
Diarrhea	8 (13.8)	5 (17.9)	3 (10.0)	0.386
Anorexia	20 (34.5)	10 (35.7)	10 (33.3)	0.849

Data are shown as *n* (%).

Abbreviation: COVID-19, coronavirus disease 2019.

On admission, most of the patients had abnormal liver function tests within 1–2 × upper limit of normal (ULN). GGT was the most common abnormality observed in the current study and 20 of the 58 patients (34.5%) had elevated GGT levels. Seventeen patients (29.3%) displayed elevated ALT levels and 13 patients (22.4%) exhibited increased AST levels.

Next, we compared the clinical features of patients with COVID-19 who had abnormal liver function tests to those of patients with COVID-19 who had normal liver function tests. We found that abnormal liver function was significantly enriched in the male and diabetes patients (Table 1, Table 2), with *P*-values of 0.004 and 0.036, respectively. No significant differences were found with regard to the other underlying diseases in patients with COVID-19 with and without abnormal liver function, including coronary artery disease, cerebrovascular disease, kidney disease, and malignancy. Also, there was no statistically significant difference in the prevalence of symptoms at illness onset between the two groups. Based on the hypothesis that the coagulation function of the participants of this study might have been impaired upon liver injury, we examined the coagulation function of each of the participants in both groups. We found that patients with COVID-19 with abnormal liver function had significantly higher D-dimer levels (Table 1, 1.45 mg/L *vs*. 0.38 mg/L, *P* <0.001).

### 3.2 Comparison between the mild and severe cases

To shed light on the link between disease progression and serum liver enzymes, we compared the levels of serum biomarkers between the mild and severe groups (Table 3). Through blood tests, we found that GGT displayed significantly higher changes in severe cases than in mild cases (71 *vs*. 23 U/L, *P* = 0.004). Despite the fact that the serum levels of other liver enzymes like ALT, ALP, TBIL, and DBIL were increased in severe cases, only the increase in the serum level of DBIL was statistically significant (*P* = 0.029).

**Table 3:** Liver functions of COVID-19 patients by the presence of severe pneumonia (*N* = 58).

Variable	Without severe pneumonia (*n* = 46)	With severe pneumonia (*n* = 12)	*P*-value
Liver functions			
AST (U/L)	21 (18–26)	19 (16–45)	0.870
ALT (U/L)	19 (12–35)	29 (15–54)	0.091
GGT (U/L)	23 (14–43)	71 (29–94)	0.004
ALP (U/L)	63 (52–72)	78 (56–86)	0.080
TBIL (μmol/L)	8.6 (6.1–12.4)	10.8 (8.0–19.5)	0.137
DBIL (μmol/L)	3.6 (2.4–5.2)	4.9 (3.7–8.0)	0.029
Albumin (g/L)	37.8 (34.9–40.5)	33.4 (31.1–38.9)	0.107
Coagulation functions			
PT (s)	13.3 (13.0–14.2)	13.8 (13.4–14.3)	0.356
PT-INR	1.02 (0.99–1.11)	1.07 (1.03–1.12)	0.321
APTT (s)	38.6 (36.0–42.0)	37.0 (24.8–40.2)	0.265
D-dimer (mg/L)	0.91 (0.29–1.89)	1.17 (0.42–8.10)	0.147
Cardiac biomarkers			
hs-cTNI (ng/L)	4.6 (1.9–8.7)	10.8 (3.7–16.6)	0.048
NT-proBNP (ng/L)	118 (58–363)	267 (83–660)	0.287
Inflammatory markers^a^			
hs-CRP (mg/L)	5.4 (1.1–20.2)	17.2 (11.8–72.3)	0.025
IL-6 (ng/L)	3.36 (1.67–9.91)	11.96 (1.94–14.24)	0.134
TNF-α (ng/L)	6.7 (5.3–10.8)	11.4 (9.1–13.2)	0.096
Procalcitonin^b^ (μg/L)	0.07 (0.05–0.09)	0.10 (0.07–0.13)	0.092

Data are shown as median (IQR).

Abbreviations: ALP, alkaline phosphatase; ALT, alanine aminotransferase; APTT, activated partial thromboplastin time; AST, aspartate aminotransferase; COVID-19, coronavirus disease 2019; DBIL, direct bilirubin; GGT, gamma-glutamyl transferase; hs-CRP, high sensitive C-reactive protein; IL-6, interleukin-6; INR, international normalized ratio; IQR, interquartile range; NT-proBNP, N-terminal pro brain natriuretic peptide; PT, prothrombin time; TBIL, total bilirubin; TNF-α, tumor necrosis factor-alpha.

a Data available for 46 patients. Thirty-six without severe pneumonia; and 10 with severe pneumonia.

b Data available for 53 patients. Forty-one without severe pneumonia; and 12 with severe pneumonia.

Besides, we also found that the serum levels of the cardiac biomarker, hs-cTNI, and the inflammatory biomarker, hs-CRP, were significantly increased in severe cases, with *P*-values of 0.048 and 0.025, respectively.

### 3.3 Duration of hospitalization in patients with abnormal liver function tests

**Fig. 1:**
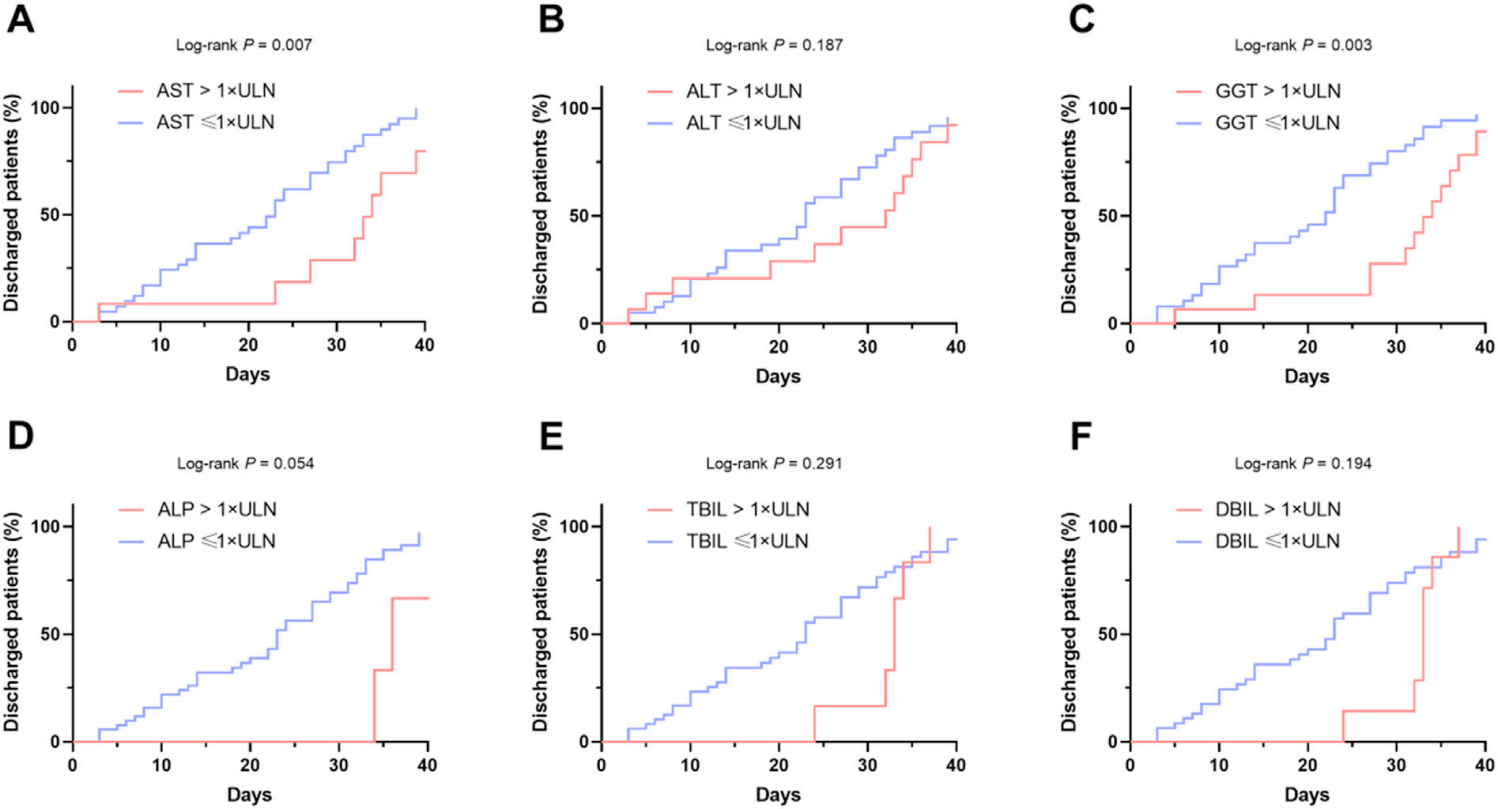
**Kaplan–Meier curves for duration of hospital stay in patients with COVID-19 with or without elevated liver enzymes.** The duration of hospital stay of patients with COVID-19 with and without 1 × ULN of **(A)** AST; **(B)** ALT; **(C)** GGT; **(D)** ALP; **(E)** TBIL; **(F)** DBIL. Abbreviations: ALP, alkaline phosphatase; ALT, alanine aminotransferase; AST, aspartate aminotransferase; COVID-19, coronavirus disease 2019; DBIL, direct bilirubin; GGT, gamma-glutamyl transferase; TBIL, total bilirubin; ULN, upper limit of normal.

We then compared the duration of hospital stay in patients with liver enzyme abnormalities. We found that elevated AST and GGT were positively correlated with longer hospitalizations with a cutoff of >1 × ULN (Fig. 1). Similar results were found with the cutoff of >2 × ULN (data not shown), suggesting that AST and GGT might be associated with disease severity.

### 3.4 GGT was a putative biomarker of disease severity

**Fig. 2:**
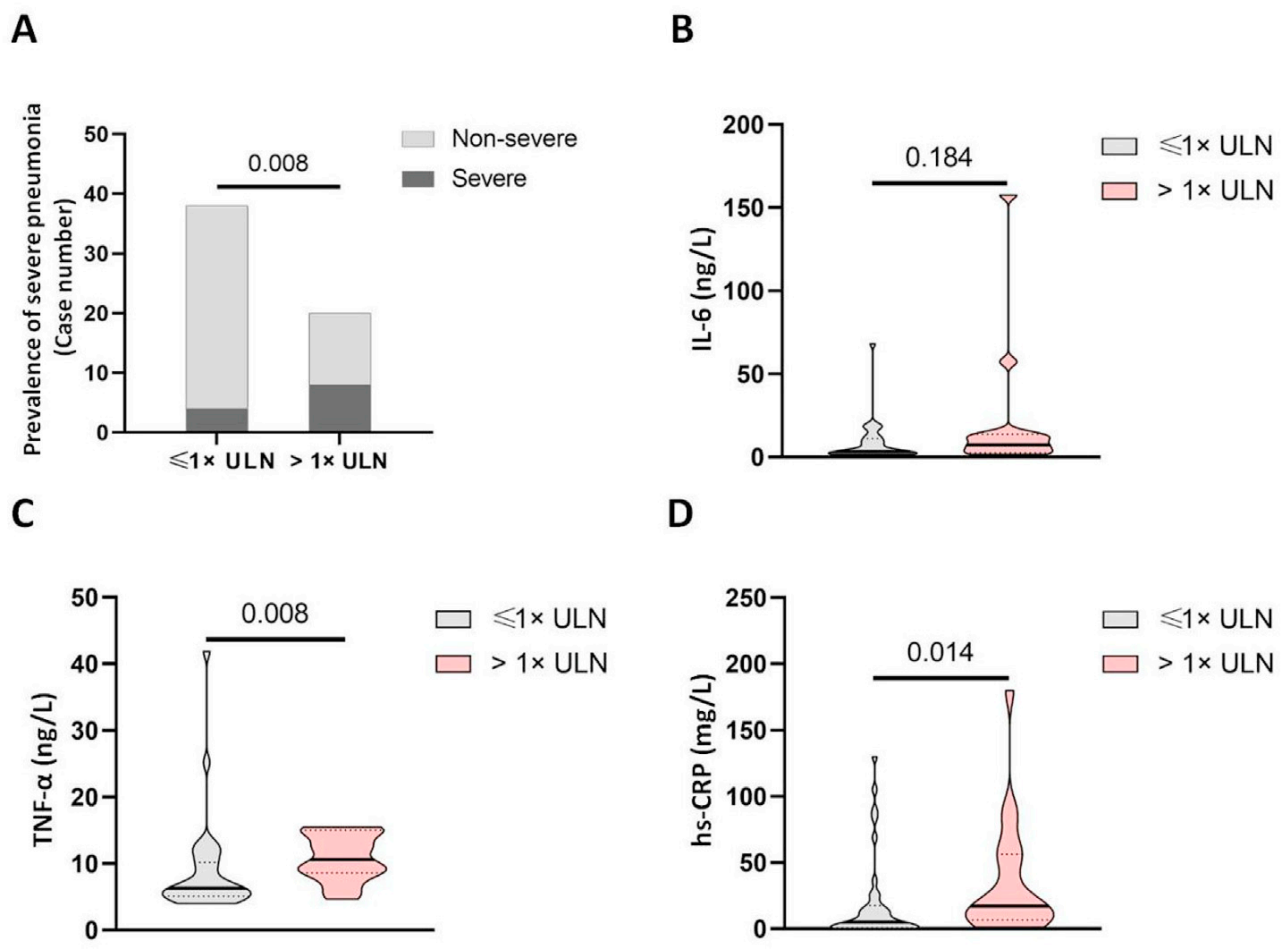
**Levels of circulating proinflammatory cytokines in patients with COVID-19 with and without elevated GGT. (A)** The distribution of mild and severe cases in patients with COVID-19 with and without elevated GGT. The levels of **(B)** IL-6, **(C)** TNF-α, and **(D)** hs-CRP in patients with COVID-19 with and without elevated GGT. Abbreviations: COVID-19, coronavirus disease 2019; hs-CRP, high sensitive C-reactive protein; IL-6, interleukin-6; TNF-α, tumor necrosis factor-alpha; ULN, upper limit of normal.

We found that serum GGT levels were significantly increased in severe cases than in mild cases, and its elevation was associated with prolonged hospital stay. We found that patients with elevated GGT had a higher risk of being severe cases (Fig. 2A). Moreover, patients with elevated GGT also had higher levels of IL-6 and hs-CRP but not TNF-α (Fig. 2B–D), suggesting that GGT might be a potent indicator of disease severity.

### 3.5 The expression patterns of GGT and ACE2 in human tissues

In terms of the important clinical implication of GGT in COVID-19, to shed light on the underlying mechanisms, we conducted bioinformatics analysis to investigate why GGT was up-regulated.

**Fig. 3:**
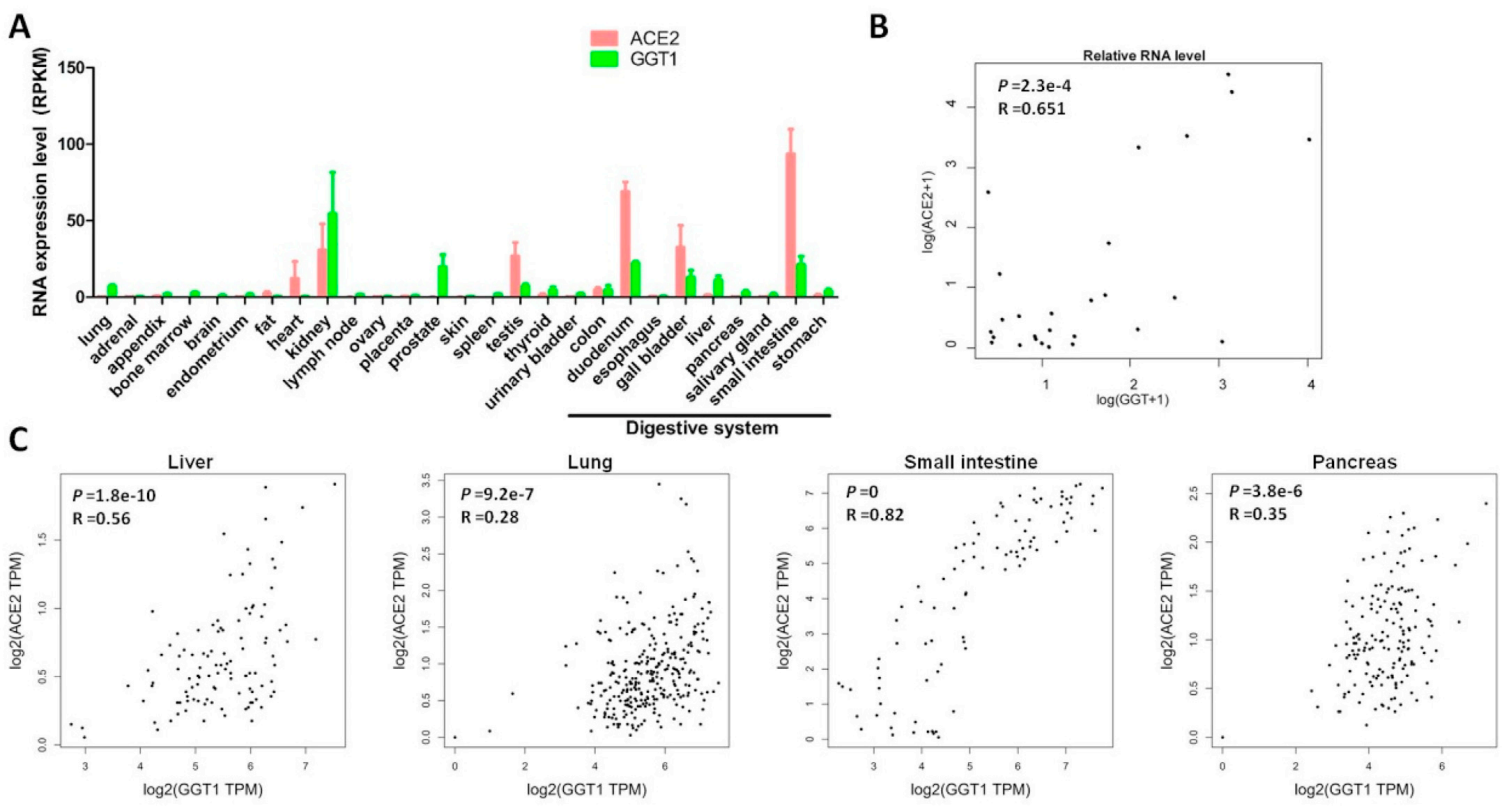
**The consistent expression of ACE2 and GGT in normal human tissues.(A)** The RNA levels of ACE2 and GGT in a panel of normal human tissues. **(B)** The correlation between ACE2 and GGT in a panel of normal human tissues. **(C)** The correlation between ACE2 and GGT in different tissues. Abbreviations: ACE2, angiotensin-converting enzyme 2; GGT, gamma-glutamyl transferase.

The increase in the serum GGT level is possibly due to the release of the enzyme from the cells attacked by the SARS-CoV-2 virus. As the distribution of the SARS-CoV-2 entry receptor ACE2 may indicate the target cells in the human body, we evaluated the expression of ACE2 and GGT in the normal human tissues. It is known that GGT1 is the dominant form of GGT.^17^ Thus, we chose GGT1 for further analysis. Interestingly, the expression pattern of ACE2 was similar to that of GGT1 in a panel of human tissues (Fig. 3A and B; Supplementary Fig. 1). Moreover, we further examined the correlation between ACE2 and GGT1 in different organs. The correlation study revealed that the expression of ACE2 was positively correlated with that of GGT in a wide variety of human tissues (Fig. 3C).

Given that the consistent expression profiles of ACE2 and GGT1 in normal human tissues, we also evaluated their potential regulators. GEPIA is a novel interactive web server for analyzing the RNA sequencing data of tumors or normal samples from the TCGA and the GTEx projects. Thus, we used GEPIA to analyze the expression profiles of ACE2 and GGT1 in human tissues. According to the data on the GEPIA website, we compared the top 100 genes that were positively associated with ACE2 and GGT1. Interestingly, we found that the expression of HNF1B was positively associated with that of ACE2 and GGT. Based on the previous ChIP-seq data, we found that HNF1B physically interacted with the promoter region of ACE2 and GGT1 (Supplementary Fig. 2A). This finding was partially reported in a previous study, showing that HNF1B and HNF1A transcriptionally activated the gene expression of ACE2 in humans and mice.^18^ Subsequent analysis found that the expression of HNF1B positively correlated with that of ACE2 and GGT1 (Supplementary Fig. 2B).

## 4 Discussion

In the current finding, we identified a previously unknown role of GGT in COVID-19. We demonstrated that serum GGT levels were significantly higher in patients with severe pneumonia than in those with mild pneumonia, the elevation of which was positively associated with longer hospital stay and disease severity. Furthermore, we found that GGT might indicate the *in vivo* expression level of ACE2 because they shared the same transcriptional machinery. Taken together, GGT may be a potential biomarker that indirectly indicates the *in vivo* level of ACE2.

Currently, the pathological impact of COVID-19 on the liver is still largely unknown.^19^^,^^20^ In our current study, we found that 51.7% of patients on admission had abnormal liver function. Previous investigations have proposed that COVID-19 triggers hepatic injury by inducing viral hepatitis. However, the derangement of the liver function is clearly mild. Thus, we cannot rule out other alternative explanations. For instance, because the lung is the major organ attacked by the SARS-CoV-2 virus and shortness of breath is common in COVID-19 patients, it is possible that hypoxia could induce liver damage. On the other hand, drug-induced liver injury is a possible contributing factor based on a recent study. It was reported that the use of lopinavir/ritonavir was associated with a higher risk of liver injury.^16^ In addition, the elevation of inflammatory factors is common in COVID-19 patients, and some of these cytokines may be detrimental to hepatocytes. Thus, more studies are needed to further elucidate the pathological impact of SARS-CoV-2 on liver injury. Moreover, the surveillance of liver enzymes during hospitalization is necessary for patients with COVID-19.

Currently, due to the absence of a large-scale epidemiological study, the population of people who are susceptible to SARS-CoV-2 infection remains unknown. It has been demonstrated that ACE2 expression correlates with the susceptibility to SARS-CoV infection,^21^ the link between ACE2 expression and the susceptibility to SARS-CoV-2 infection is still to be determined. A recent retrospective study analyzed 425 confirmed COVID-19 cases and found that only a few of the patients were children.^22^ This finding might result from the different expression profiles of ACE2 in children and adults. Interestingly, GGT displayed an age-related profile in humans and usually, adults have higher levels of GGT.^23^ Nevertheless, there is insufficient evidence to demonstrate that ACE2 expression varies with age, and further investigations are needed to address this question.

Another concern is that the higher ACE2 level might indicate aggravated health conditions due to a higher viral load in the human body. A recent study found that the cells with higher levels of ACE2 usually harbored enhanced pseudotype SARS-CoV-2 entry and virus titer.^24^ A previous transgenic animal model demonstrated that enhanced expression of human ACE2 accelerated disease severity in mice infected with SARS-CoV, suggesting that ACE2-dependent viral entry into cells is essential for disease progression.^25^ Moreover, it was reported that higher initial viral load was associated with a worse prognosis in the SARS patients and a recent study found that severe COVID-19 cases tend to harbor higher virus titers than mild cases,^26^^,^^27^ suggesting that a higher viral load might be a potent marker for assessing disease severity. In the current study, we found that elevated levels of GGT were exhibited in severe COVID-19 cases. Similar findings were also reported by another independent group.^28^ Since ACE2 may utilize the same regulator network as GGT, they have consistent expression profiles across various human tissues. It is possible that patients with increased GGT levels may have higher levels of ACE2, leading to a higher viral load in target cells and worse health conditions. In addition, this finding also raised a question about the impact of pre-existing liver disease on COVID-19. For instance, if a patient with pre-existing disease has an elevated GGT level, will this patient be more vulnerable to SARS-CoV-2 infection?

Our study had some limitations. The number of study participants was small and the study was limited to a single center. Therefore, more studies with larger samples carried out at multiple centers are needed to comprehensively clarify the hepatic function impairment and the clinical importance of GGT in COVID-19 patients. Nevertheless, we believe that our current results offered a pilot clue of liver and bile duct injury mediated by COVID-19.

In conclusion, we demonstrated a previously unknown role of GGT in COVID-19 patients. Increased GGT levels were common in severe cases, and its elevation was positively correlated with disease severity and a longer hospital stay. Due to the consistent expression with ACE2, GGT may serve as a biomarker that indicates the susceptibility of SARS-CoV-2 infection.

## Authors’ contributions

J. Liu, C. Yu and Q. Yang contributed equally to this work. Conceiving and designing the experiments: Q. Yang, J. Liu, W. Liang, C. Yu, G. Chen and Y. Yang. Case collection: Q. Yang, J. Liu, X. Yuan and F. Yang. Data extraction: F. Yang and P. Li. Statistical analysis: C. Yu. Interpretation of results: C. Yu, W. Liang and Y. Yang. Writing and revising paper: C. Yu, W. Liang and Y. Yang.

## Declaration of competing interest

The authors declare that they have no conflict of interest.

## References

[1] Guan W.J., Ni Z.Y., Hu Y. (2020). Clinical characteristics of coronavirus disease 2019 in China. N Engl J Med.

[2] Huang C., Wang Y., Li X. (2020). Clinical features of patients infected with 2019 novel coronavirus in Wuhan, China. Lancet.

[3] Chen N., Zhou M., Dong X. (2020). Epidemiological and clinical characteristics of 99 cases of 2019 novel coronavirus pneumonia in Wuhan, China: a descriptive study. Lancet.

[4] Zhou F., Yu T., Du R. (2020). Clinical course and risk factors for mortality of adult inpatients with COVID-19 in Wuhan, China: a retrospective cohort study. Lancet.

[5] Benedé-Ubieto R., Estévez-Vázquez O., Flores-Perojo V. (2021). Abnormal liver function test in patients infected with coronavirus (SARS-CoV-2): a retrospective single-center study from Spain. J Clin Med.

[6] Zhang C., Shi L., Wang F.S. (2020). Liver injury in COVID-19: management and challenges. Lancet Gastroenterol Hepatol.

[7] Chau T.N., Lee K.C., Yao H. (2004). SARS-associated viral hepatitis caused by a novel coronavirus: report of three cases. Hepatology.

[8] Peiris J.S., Lai S.T., Poon L.L. (2003). Coronavirus as a possible cause of severe acute respiratory syndrome. Lancet.

[9] Alsaad K.O., Hajeer A.H., Al Balwi M. (2018). Histopathology of Middle East respiratory syndrome coronovirus (MERS-CoV) infection - clinicopathological and ultrastructural study. Histopathology.

[10] Lei C., Qian K., Li T. (2020). Neutralization of SARS-CoV-2 spike pseudotyped virus by recombinant ACE2-Ig. Nat Commun.

[11] Li W., Moore M.J., Vasilieva N. (2003). Angiotensin-converting enzyme 2 is a functional receptor for the SARS coronavirus. Nature.

[12] Liang W., Feng Z., Rao S. (2020). Diarrhoea may be underestimated: a missing link in 2019 novel coronavirus. Gut.

[13] Zou X., Chen K., Zou J., Han P., Hao J., Han Z. (2020). Single-cell RNA-seq data analysis on the receptor ACE2 expression reveals the potential risk of different human organs vulnerable to 2019-nCoV infection. Front Med.

[14] Boeckmans J., Rodrigues R.M., Demuyser T., Piérard D., Vanhaecke T., Rogiers V. (2020). COVID-19 and drug-induced liver injury: a problem of plenty or a petty point?. Arch Toxicol.

[15] Naicker S., Yang C.W., Hwang S.J., Liu B.C., Chen J.H., Jha V. (2020). The Novel Coronavirus 2019 epidemic and kidneys. Kidney Int.

[16] Cai Q., Huang D., Yu H. (2020). COVID-19: abnormal liver function tests. J Hepatol.

[17] Courtay C., Heisterkamp N., Siest G., Groffen J. (1994). Expression of multiple gamma-glutamyltransferase genes in man. Biochem J.

[18] Pedersen K.B., Chhabra K.H., Nguyen V.K., Xia H., Lazartigues E. (2013). The transcription factor HNF1α induces expression of angiotensin-converting enzyme 2 (ACE2) in pancreatic islets from evolutionarily conserved promoter motifs. Biochim Biophys Acta.

[19] Bangash M.N., Patel J., Parekh D. (2020). COVID-19 and the liver: little cause for concern. Lancet Gastroenterol Hepatol.

[20] Zhao X., Lei Z., Gao F., Xie Q., Jang K., Gong J. (2021). The impact of coronavirus disease 2019 (COVID-19) on liver injury in China: a systematic review and meta-analysis. Medicine (Baltimore).

[21] Hofmann H., Geier M., Marzi A. (2004). Susceptibility to SARS coronavirus S protein-driven infection correlates with expression of angiotensin converting enzyme 2 and infection can be blocked by soluble receptor. Biochem Biophys Res Commun.

[22] Li Q., Guan X., Wu P. (2020). Early transmission dynamics in Wuhan, China, of novel coronavirus-infected pneumonia. N Engl J Med.

[23] Bussler S., Vogel M., Pietzner D. (2018). New pediatric percentiles of liver enzyme serum levels (alanine aminotransferase, aspartate aminotransferase, γ-glutamyltransferase): effects of age, sex, body mass index, and pubertal stage. Hepatology.

[24] Hoffmann M., Kleine-Weber H., Schroeder S. (2020). SARS-CoV-2 cell entry depends on ACE2 and TMPRSS2 and is blocked by a clinically proven protease inhibitor. Cell.

[25] Yang X.H., Deng W., Tong Z. (2007). Mice transgenic for human angiotensin-converting enzyme 2 provide a model for SARS coronavirus infection. Comp Med.

[26] Chu C.M., Poon L.L., Cheng V.C. (2004). Initial viral load and the outcomes of SARS. CMAJ.

[27] Liu Y., Yan L.M., Wan L. (2020). Viral dynamics in mild and severe cases of COVID-19. Lancet Infect Dis.

[28] Shao T., Tong Y., Lu S. (2020). γ-Glutamyltransferase elevations are frequent in patients with COVID-19: a clinical epidemiologic study. Hepatol Commun.

