# Supplementary material for: The clinical implication of gamma-glutamyl transpeptidase in COVID-19
Source: Liver Res. 2021 Sep 25;5(4):209–16. doi: 10.1016/j.livres.2021.09.001 (PMC8464026; doi:10.1016/j.livres.2021.09.001)
Supplement: Multimedia component 1 [file mmc1.docx]

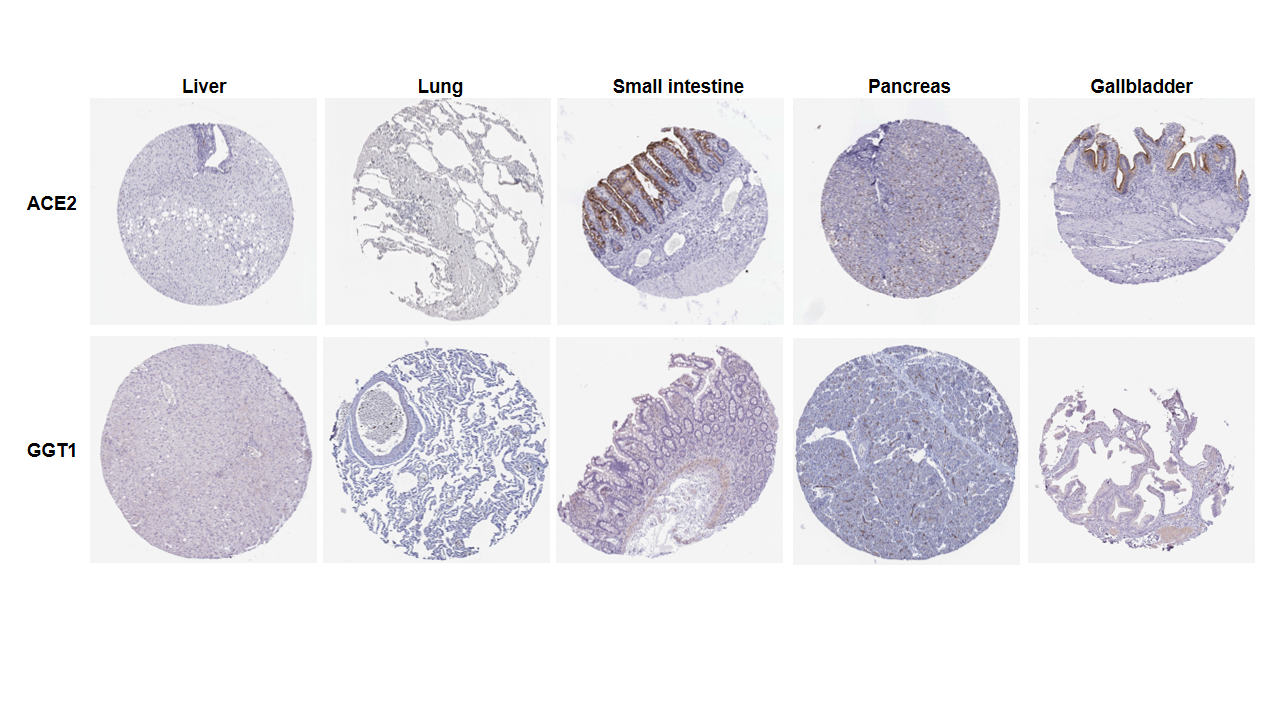


**Supplementary Fig. 1.** **The protein levels of GGT1 and ACE2 in different human tissues.** The expression patterns of GGT1 and ACE2 were obtained from The Human Protein Atlas website. Abbreviations: ACE2, angiotensin-converting enzyme 2; GGT, gamma-glutamyl transferase.

Given that the consistent expression profiles of ACE2 and GGT1 in normal human tissues, we also evaluated their potential regulators. GEPIA is a novel interactive web server for analyzing the RNA sequencing data of tumors or normal samples from the TCGA and the GTEx projects. Thus, we used GEPIA to analyze the expression profiles of ACE2 and GGT1 in human tissues. According to the data on the GEPIA website, we compared the top 100 genes that were positively associated with ACE2 and GGT1. Interestingly, we found that the expression of HNF1B was positively associated with that of ACE2 and GGT. Based on the previous ChIP-seq data, we found that HNF1B physically interacted with the promoter region of ACE2 and GGT1 (Supplementary Fig. 2A). This finding was partially reported in a previous study, showing that HNF1B and HNF1A transcriptionally activated the gene expression of ACE2 in humans and mice.^18^ Subsequent analysis found that the expression of HNF1B positively correlated with that of ACE2 and GGT1 (Supplementary Fig. 2B).


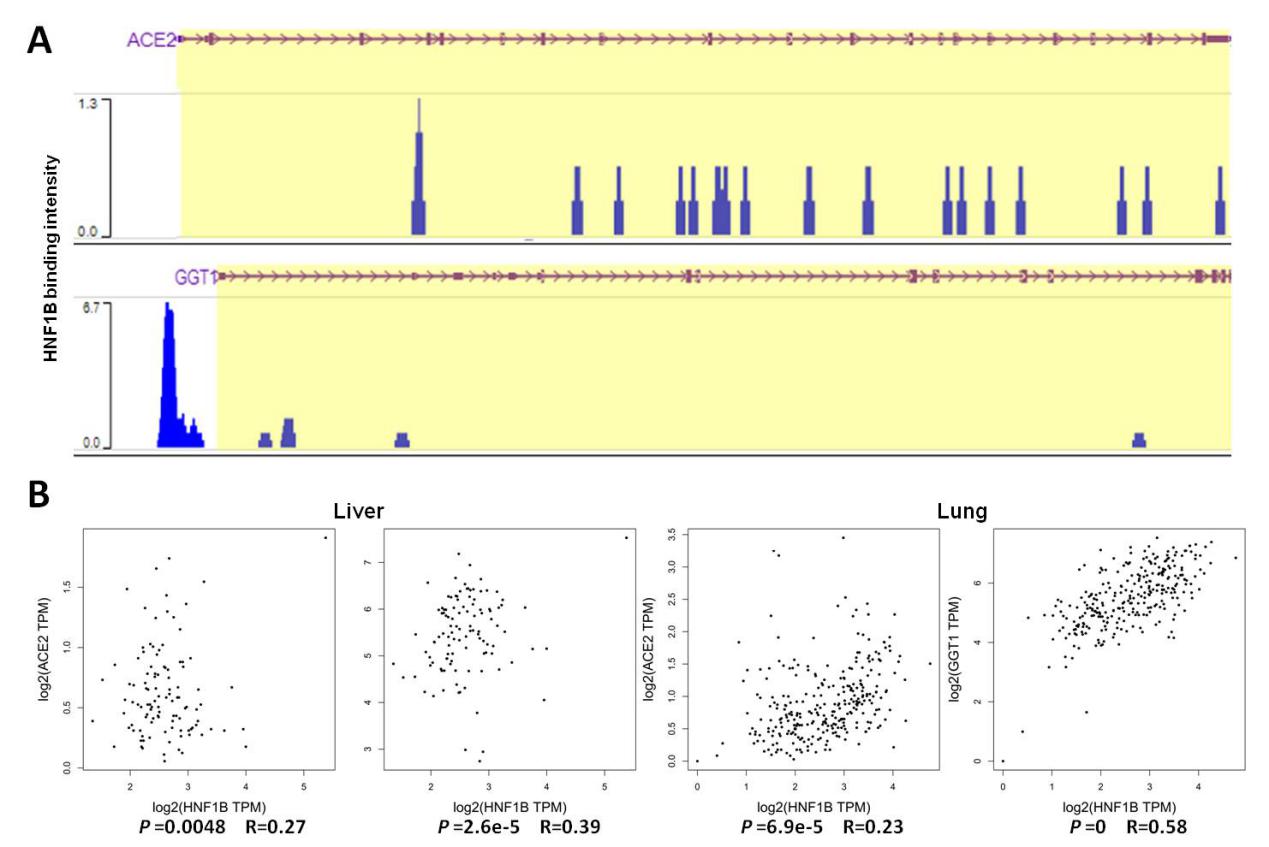


**Supplementary Fig. 2.** **Both GGT1 and ACE2 were transcriptionally regulated by HNF1B.** **(A)** The ChIP-seq data showed that HNF1B was bound to the promoter region of ACE2 and GGT in human cells. **(B)** The correlation between HNF1B, ACE2, and GGT in human liver and lung tissues. Abbreviations: ACE2, angiotensin-converting enzyme 2; ChIP-seq, chromatin immunoprecipitation sequencing; GGT, gamma-glutamyl transferase; HNF1B, hepatic nuclear factor-1β.
